# Diagnostic Value of FDG PET-CT Quantitative Parameters and Deauville-Like 5 Point-Scale in Predicting Malignancy of Focal Thyroid Incidentaloma

**DOI:** 10.3389/fmed.2019.00024

**Published:** 2019-02-12

**Authors:** Philippe Thuillier, David Bourhis, Nathalie Roudaut, Geneviève Crouzeix, Zarrin Alavi, Ulrike Schick, Philippe Robin, Véronique Kerlan, Pierre-Yves Salaun, Ronan Abgral

**Affiliations:** ^1^Department of Endocrinology, University Hospital of Brest, Brest, France; ^2^EA GETBO 3878, University Hospital of Brest, Brest, France; ^3^Department of Nuclear Medicine, University Hospital of Brest, Brest, France; ^4^INSERM CIC-1412 Medical University Hospital of Brest, Brest, France; ^5^Department of Radiotherapy, University Hospital of Brest, Brest, France

**Keywords:** focal thyroid incidentaloma, positron emission tomography computed tomography, metabolic tumor volume, tumor lesion glycolysis, SUVmax, quantitative PET parameters

## Abstract

**Objective:** To evaluate the diagnostic value of FDG PET-CT metabolic parameters and Deauville-like 5 point-scale to predict malignancy in a population of patients presenting focal thyroid incidentaloma (fTI).

**Design:** This retrospective study included 41 fTI, classified according to cytological and histological data as benign (BL) or malignant lesion (ML). FDG PET-CT semi-quantitative parameters (SUVmax, SUVmean, SUVpeak, MTV, TLG), tumor to liver SUVmean ratio (TLRmax and TLRmean), tumor to blood-pool SUVmean ratio (TBRmax and TBRmean) were calculated. Each fTI was also classified on a Deauville-like 5-point scale (DS) currently used in lymphoma. Comparison between BL and ML was performed for each parameter and a ROC analysis was conducted.

**Results:** All quantitative PET metabolic parameters (SUV parameters, volume based parameters and SUV ratio) were higher in ML compared with BL, yet no significant difference was reported. fTI (uptake) malignancy rate according to DS grades 2, 3, 4, and 5 was, respectively, 25% (1 of 4), 28.6% (2 of 7), 8.3% (1 of 12), and 33.3% (6 of 18) with no significant difference between ML and BL groups. Results of ROC analysis showed that mean TBR had the highest AUC in our cohort (0.66 95%CI [0.41; 0.91]) with a cut-off value of 2.2. Specificity of MTV and TLG was 100% (cut-off values: MTV 9.6 ml, TLG 22.9 g) and their sensitivity was 30 and 40%, respectively.

**Conclusion:** Our study did not highlight any FDG PET/CT parameter predictor of fTI malignancy.

## Introduction

Thyroid incidentaloma (TI) is a thyroid lesion fortuitously detected in patients undergoing an imaging for a non-thyroid purpose. The common use of 18F-fluorodeoxyglucose (FDG) positron emission tomography—computed tomography (PET-CT) in the field of oncology leads to a whole body imaging allowing the discovery of unexpected lesions. In FDG PET-CT, TI may appear as a focal FDG uptake (fTI) or as diffuse thyroid uptake (dTI) of the thyroid parenchyma. Recently, we published a prospective cohort study conducted on a population of 10,118 patients undergoing FDG PET-CT. Our previous results highlighted a fTI prevalence of 1.3% and a malignancy rate of 16.6% (1). This prevalence was consistent with the literature whereas our malignancy rate was lower than that reported by recent meta-analyses (2, 3).

Currently, due to the lack of evidence on optimal management of fTI, ATA (American Thyroid Association) guidelines proposed to perform a fine needle aspiration biopsy (FNAB) in all fTI >1 cm (4) but this approach remains not fully supported. There is a paucity of literature with quality clinical evidence exploring the current guidelines. Recent literature has confirmed the interest of ultrasound classifications (3–7) in management of fTI. Yet there is a need for additional predictors of malignancy to avoid unnecessary operations.

Maximal standardized uptake value (SUVmax) in FDG PET-CT has been widely assessed in the literature to predict malignancy in fTI. Some studies reported higher SUVmax value in benign (BL) vs. malignant (ML) fTI (8, 9), while others shown no statistical difference. In our previous study (1), median SUVmax was higher (10.4 vs. 6.4) in malignant than in benign fTI groups but without significance (*p* = 0.649). These discrepant SUVmax results can be explained by the differences in SUV measurement methodology, PET technology, and FDG administration procedure across the studies. Consequently, SUVmax-related cut-offs could not be compared to reach a consensual threshold for accurate differential diagnosis between benign and malignant incidentaloma (3). New quantitative PET parameters have also been proposed to overcome the SUV measurement-related disadvantages (a single pixel value within a tumor with potential heterogeneous features due to cell proliferation, necrosis, angiogenesis) (10). The latter affects both patient management and clinical study's power. Yet, there is a paucity of literature on these PET quantitative parameters. Recently, Shi et al. assessed diagnostic performance of volume-based PET parameters, Metabolic Tumor Volume (MTV) and Total Lesion Glycolysis (TLG), and showed higher MTV and TLG values in malignant lesions (11). Another recent study evaluated several SUVmax ratios as Tumor-to-Blood-pool Ratio (TBR) and Tumor-to-Liver-Ratio (TLR) and showed good diagnostic performance with AUC of 0.78 for both (12).

The objective of this ancillary study was to investigate the predictive value of different quantitative PET parameters and a “Deauville-like” 5-point scale (DS) in diagnosis of malignant fTI.

## Materials and Methods

Population and study protocol was described in our previous publication (1). Among the 92 fTI included in the princeps study (patients who underwent a US ± FNAB), only 41 fTI could be classified as benign or malignant according to cytological and histological data. Mean age ± SD of our patients (26 women, 15 men) was 61.0 ± 12.3 years old. Mean fTI diameter was 17.78 ± 10.15 mm. In the absence of histological data, classification of thyroid nodules was done according to cytological findings using Bethesda classification (2 for benign and 5 or 6 for malignant). Thyroid nodules classified 1, 3, and 4 were excluded.

We performed a retrospective and ancillary study about a cohort of patients already published in another journal (1). Ethical review and approval was not required for this study in accordance with the local legislation and institutional requirements. All the patients gave a written and informed consent for the use of their images.

### Imaging Procedures

**Figure 1:**
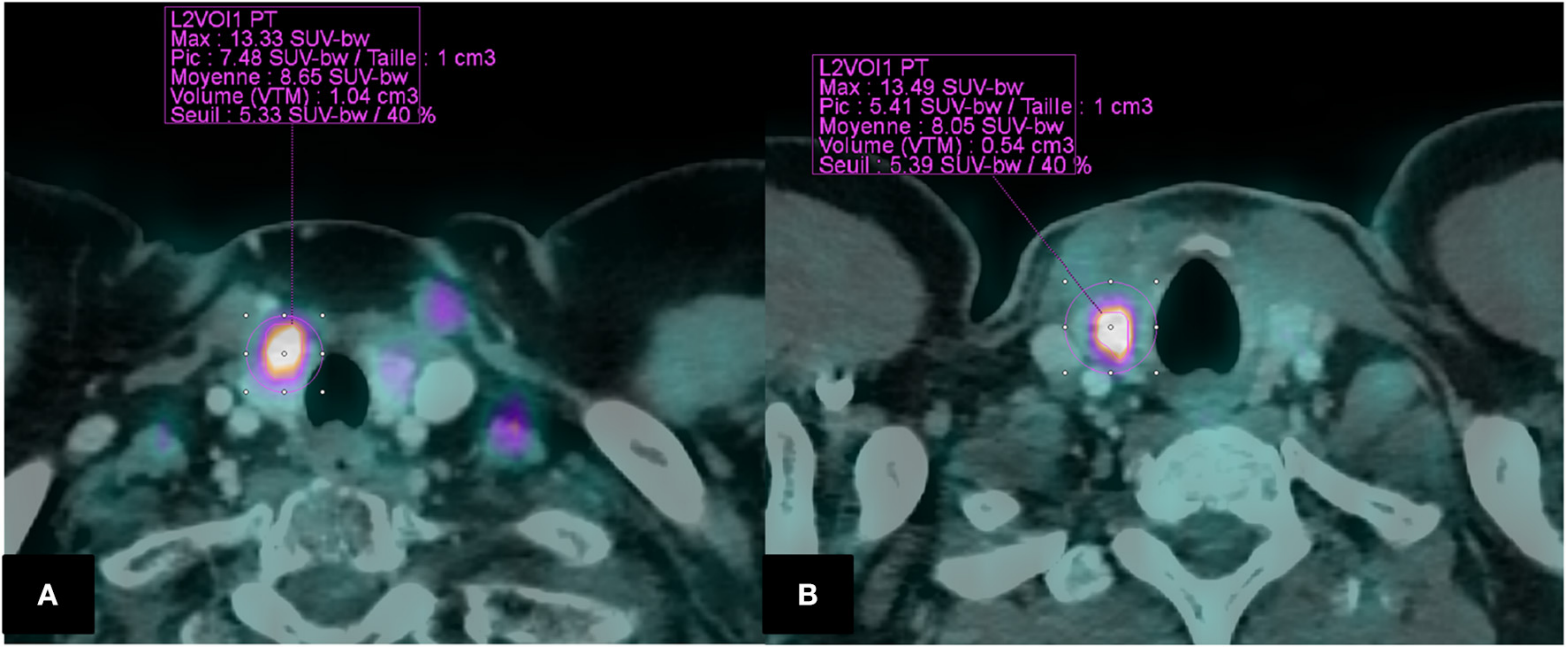
Segmentation of fTI using a fixed SUV threshold method (40 % of SUVmax) Example of two patients **(A)**: BL in a 54,F (SUVmax = 13.33; MTV = 1.04; TLG = 8.99; TBRmax = 7.15; TBRmean = 4.60; TLRmax = 4.22; TLRmean = 2.72; Deauville “like” Scale = 5). **(B)**: ML in a 38,H (SUVmax = 13.49; MTV = 0.54; TLG = 4.34. TBRmax = 7.45; TBRmean = 4.44 TLRmax = 5.81; TLRmean = 3.46; Deauville “like” Scale = 5).

FDG PET-CT scan was performed on a Biograph mCT S64 (Siemens medical, Erlangen, Germany). Patients fasted 4 h before PET acquisitions, and the blood glucose level had to be < 7 mmol/L before injection of 370 MBq (5 MBq/Kg) of FDG. A SUV-based approach was used to determinate quantitative PET parameters. All tumors were then segmented using a fixed SUV threshold method for delineating a 3D contour around voxels equal to or >40% of SUVmax (Figure 1) allowing to calculate PET metabolic parameters (SUVmax, SUVmean, SUVpeak), volume-based parameters [MTV and TLG (TLG = SUVmean × MTV)]. The fTI SUVmax and SUVmean were corrected from liver SUV mean, measured in a 5 cm diameter ROI placed on the right lobe to calculate tumor-to-liver ratios (TLRmax and TLRmean, respectively) and from blood pool SUVmean, measured in the aortic arch lumen to calculate tumor-to-blood-pool ratios (TBRmax and TBRmean, respectively). Additionally, fTI FDG uptake was also graded according to Deauville scale (DS) as previously reported in lymphoma PET assessment (13): (1): no uptake; (2): uptake ≤ mediastinum uptake; (3): uptake > mediastinum but ≤ liver uptake; (4): slightly higher than liver uptake; (5): markedly higher than liver uptake.

### Statistics

The Fisher exact and Mann–Whitney *U*-tests were used to make comparisons between groups as appropriate.

For each parameter, diagnostic performance in discriminating between BL and ML was assessed with a ROC analysis and the best cut-off point in each parameter was determined by the Youden index (14). Area under the curve (AUC) (*p*-value was calculated for testing AUC = 0.5), sensitivity, specificity, and accuracy were reported.

All analyses were conducted at the 0.05 significance level using XLSTAT® software (Addinsoft, Paris, France).

## Results

Median values of all quantitative PET metabolic parameters (SUVs, volume based parameters and ratios) were higher in ML than in BL group but without statistical significance (Table 1).

**Table 1:** PET quantitative parameters and 5-point Scale in BL and ML.

**PET parameters**	**Benign (*n* = 31)**	**Malignant (*n* = 10)**	***p*-value**
SUVmax	6.5 (5.2–12.9)	10.4 (4.5–12.9)	0.649
MTV	2.1 (1.0–3.6)	2.2 (0.5–12.4)	0.748
SUVmean	4.0 (3.0–8.6)	6.6 (4.0–8.0)	0.335
SUVpeak	4.4 (3.4–5.4)	5.0 (3.3–7.3)	0.45
TLG	8.5 (5.9–11.6)	10.3 (5.3–51.3)	0.235
TBRmax	3.9 (3.0–7.1)	7.1 (4.5–7.7)	0.167
TBRmean	2.2 (1.8–4.4)	4.2 (2.8–5.2)	0.131
TLRmax	3.1 (2.4–5.3)	4.5 (3.3–5.7)	0.234
TLRmean	1.7 (1.4–3.2)	3 (2.1–3.4)	0.26
5-point Scale			0.795
2 ou 3	8 (26%)	3 (30%)	
4 ou 5	23 (74%)	7 (70%)	

Continuous variables were summarized as median (interquartile range).

*Categorical variables were presented as n = (%)*.

fTI malignancy rate according to DS grades 2, 3, 4, and 5 was, respectively, 25% (1 of 4), 28.6% (2 of 7), 8.3% (1 of 12), and 33.3% (6 of 18) with no significant difference between ML and BL groups. ROC analysis with AUC and diagnostic performance of PET quantitative parameters are showed in Table 2. TBRmean had the highest AUC in our cohort (0.66 CI 95% [0.41; 0.91]) with a cut-off value of 2.2 but was not significantly different from 0.5 (*p* = 0.2). Specificity of MTV and TLG was 100% (cut-off values: MTV 9.6 ml, TLG 22.9 g) and their sensitivity was 30 and 40%, respectively.

**Table 2:** Areas under the curve (AUC), AUC 95% confidence intervals (CI), and diagnostic performance of PET quantitative parameters.

	**Cut-off**						
**Parameters**	**AUC**	**95% CI**	***p*-value**	**value**	**Se (%)**	**Sp (%)**	**Acc (%)**
SUVmax	0.55	[0.27; 0.82]	0.73	6.5	70	54.8	58.5
MTV40%	0.53	[0.24; 0.83]	0.82	9.6	30	100	82.1
SUVmean	0.60	[0.35; 0.85]	0.42	4.3	70	58.6	61.5
SUVpeak	0.58	[0.31; 085]	0.55	4.4	70	51.7	56.4
TLG	0.61	[0.32; 0.90]	0.44	22.9	40	100	84.6
TBRmax	0.65	[0.39; 0.90]	0.26	6.8	60	72.4	69.2
TBRmean	0.66	[0.41; 0.91]	0.20	2.45	80	58.6	64.1
TLRmax	0.63	[0.37; 0.87]	0.32	3.21	80	58.6	64.1
TLRmean	0.62	[0.36; 0.88]	0.35	1.86	80	58.6	64.1

## Discussion

We investigated the diagnostic performance of FDG PET/CT to differentiate benign from malignant fTI using different PET quantitative parameters and DS grading.

Regarding the common SUV approach, our study showed higher median SUVmax in ML vs. BL group but without significance (*p* = 0.649). These results were consistent with the literature. Indeed, in a meta-analysis conducted by Bertagna et al. 16 of 19 available studies found higher SUVmax value in patients with malignant vs. benign fTI (range 3.4–14.2 vs. 2.9–8.2, respectively) (3) but only 9 highlighted a significant difference between the two groups. In another meta-analysis assessing 80 BL and 78 ML, mean SUVmax was 4.8 ± 3.1 and 6.9 ± 4.7, respectively (*p* < 0.001) confirming the overall trend of having a higher SUVmax value in ML than in BL (2). However, to reach an optimal SUV cut-off in prediction of fTI malignancy remains controversial. Indeed, despite the reported higher SUVmax in ML, there is an overlap of SUVmax values between ML and BL.

In addition, PET/CT systems and acquisition protocols differ from one center to another thus leading to a variance in SUVmax thresholds (15, 16). For example, utilization of ToF systems and different reconstruction algorithms including point-spread function improves signal to noise ratio and reduces partial volume effect, but results in higher SUV. Consequently, the use of a reference tissue such as the liver or the blood-pool has become a well-established practice in PET for assessment of malignancy in some solid tumors aggressiveness (17).We investigated tumor-to-liver (TLR) and tumor-to-blood-pool (TBR) ratios but once again no significant difference was found. TBRmean had the best AUC value but with a confidence interval including 0.5 (0.66 [0.41–0.91]). Our median TLRmax results were consistent with those of a retrospective study (median TLRmax: 1.73 in 16 BL and 2.36 in 11 ML (*P* = NS) (18). However, a recent study showed significant higher TBRmax and TLRmax in the ML group. ROC analysis also highlighted threshold values (TBRmax = 1.9 and TLRmax = 1.5) with an AUC of 0.78 for both groups to differentiate between malignant and benign fTI (12).

MTV and TLG are volume-based parameters estimating metabolic tumor burden. These parameters have been widely assessed as predictors of prognosis in solid tumors (19–22). We found no significant difference in malignancy rate between MTV and TLG unlike both parameters showed a good specificity in ROC analysis. Few studies assessed these parameters for their diagnosis value to distinguish BL from ML in thyroid (23). Our results were consistent with a large retrospective cohort study assessing 200 fTI (23). Indeed Kim et al. showed that MTV with a relative SUVmax = 40% cut-off and TLG were similar in ML and BL: 5.76 vs. 5.00 (*p* = 0.5031) and 16.01 vs. 15.27 (*p* = 0.8655), respectively. However, another large retrospective cohort study highlighted that MTV and TLG was higher in ML vs. in BL group. They found the highest diagnostic performance in using a fixed-SUV threshold = 4.0 to delineate lesion (sensitivity, specificity and AUC value of 85.9 and 81.3%, 71.4 and 94.3%, 0.872 and 0.895, respectively). Moreover, MTV combined with SUVmax improve positive predictive value vs. each parameter alone (11). These inconsistent results can be explained by the difference in population characteristics. Firstly, it is important to underline that SUVmax in ML and BL was statistically higher in the study by Shi et al. compared with our cohort (11.3 vs. 4.8, *p* < 0.001). Secondly, our malignancy rate was similar to that of Kim et al. (24.3 and 24.5%, respectively) whereas Shi et al. reported a much higher rate (64.6%, *n* = 64/99) suggesting again a difference in studies populations. Finally, 7 anaplastic carcinoma and 8 medullary thyroid carcinoma were found among the 64 malignant lesions in Shi et al. study suggesting particular histological patterns. Our pathological results (6 papillary carcinomas and 3 follicular thyroid cancers) were comparable with those by Kim et al. (2 follicular carcinomas and 47 papillary thyroid cancers).

To our knowledge, this is the first study assessing FDG PET/CT identified fTI using a Deauville-like scale (DS) approach for interpretation of malignancy. DS is a simple and reproducible scale based on visual analysis and widely used for intermediate therapeutic assessment in patients with diffuse large B cell lymphoma and Hodgkin lymphoma (13). Unfortunately, DS grading of FDG PET/CT identified fTI was not statistically significant between ML and BL groups.

Our study has several limitations. Firstly, it was a single-center study. Secondly, only a small number of fTI were identified and assessed (i.e., 41 lesions in compliance with the gold standard criteria). This lack of statistical power can explain our non-significant results in prediction of fTI malignancy between the different PET/CT quantitative parameters. Future related multicentric studies with larger sample of fTI are warranted for conclusive results.

## Author Contributions

VK, P-YS, and RA are the guarantors of the paper. PT, DB, and RA designed the study. PT realized statistics. PT drafted the manuscript. PT, NR, GC, ZA, US, PR, and RA did interpretation of data. NR, ZA, PR, US, and RA revised the manuscript for intellectual content. All authors contributed in drawing up the manuscript.

### Conflict of Interest Statement

The authors declare that the research was conducted in the absence of any commercial or financial relationships that could be construed as a potential conflict of interest.
